# Prognostic value of neutrophil to lymphocyte ratio in acute ischemic stroke after reperfusion therapy

**DOI:** 10.1038/s41598-021-85373-5

**Published:** 2021-03-17

**Authors:** Ying Bi, Jing Shen, Sheng-Cai Chen, Ji-Xiang Chen, Yuan-Peng Xia

**Affiliations:** Department of Neurology, Union Hospital, Tongji Medical College, Huazhong University of Science and Technology, Wuhan, 430022 China

**Keywords:** Neurology, Neurological disorders

## Abstract

The purpose of this study was to investigate whether baseline neutrophil to lymphocyte ratio (NLR) was an independent predictor for early symptomatic intracranial hemorrhage (sICH), poor functional outcome and mortality at 3 months after reperfusion therapy in acute ischemic stroke (AIS) patients. Using PubMed and EMBASE, we searched for literature published before January 19th, 2019. Two reviewers independently confirmed each study’s eligibility, assessed risk of bias, and extracted data. One reviewer combined studies using random effects meta-analysis. 9 studies with 3651 patients were pooled in the meta-analysis. Overall, baseline NLR levels were greater in patients with poor outcome. The standardized mean difference (SMD) in the NLR levels between patients with poor functional outcome (mRS > 2) and good functional outcome (mRS ≤ 2) was 0.54 units (95% credible interval [CI] [0.38, 0.70]). Heterogeneity test showed that there were significant differences between individual studies (p = 0.02; I^2^ = 72.8%). The NLR levels were associated with sICH in four included studies (n = 2003, SMD = 0.78, 95% [CI] [0.18, 1.38], I^2^ = 73.9%). Higher NLR levels were positively correlated with 3-month mortality (n = 1389, ES = 1.71, 95% CI [1.01,2.42], p < 0.01, I^2^ = 0%) when data were used as categorical variables. Our meta-analysis suggests that increased NLR levels are positively associated with greater risk of sICH, 3-month poor functional outcome and 3-month mortality in AIS patients undergoing reperfusion treatments. Although there are some deficits in this study, it may be feasible to predict the prognosis of reperfusion therapy in AIS patients with NLR levels.

## Introduction

Stroke is one of the leading causes of death worldwide and bring heavy life burden^1^. Over 85% of acute stroke are caused by cerebral ischemia and reperfusion therapy is a milestone in acute ischemic stroke (AIS) treatment. Intravenous thrombolysis (IVT) using recombinant tissue-type plasminogen activator (r-tPA) within 4.5 h and intervention with endovascular treatment (EVT) such as mechanical thrombectomy (MT), are strongly recommended for AIS treatment^2,3^. However, Symptomatic intracranial hemorrhage (sICH) is the most serious complication after IVT and results in poor outcomes and only a minority of patients benefit from such treatments^4,5^. Recent trials show that the better outcomes of EVT appear due to the strict selection of patients^6^. Early and accurate assessment of the prognosis of AIS may help neurologists to select the optimal therapeutic strategy and minimize the aftermath of ischemic stroke. Therefore, it is important to find out new biomarkers that could predict the risk of early sICH and poor outcome in AIS patients before reperfusion therapy.

Neutrophil to lymphocyte ratio (NLR) in blood routine, low cost and widely available, has been widely studied as a prognostic factor^7–10^. Numerous studies have demonstrated that higher NLR levels are associated with poor outcomes and stroke occurrence^11–14^. Several meta-analyses have indicated that elevated NLR level is a negative prognostic indicator in AIS and spontaneous intracerebral hemorrhage^11,15–18^. Furthermore, NLR has recently been recognized as a prognostic marker in patients undergoing cardiac revascularization^19,20^. Reperfusion therapy in AIS has a similar pathophysiological mechanism with coronary artery recanalization treatment and the association between NLR and outcomes of AIS with reperfusion therapy is still under discussion. In 2014, Brooks et al. performed a retrospective cohort study and the result indicated that NLR ≥ 5.9 predicted poor outcome and mortality at 90 days^21^. However, another multi-center study by Duan showed that NLR ≥ 7.0 was independently associated with poor functional outcome and no significant influence was found between NLR level and 3-month mortality^22^. This may be due to the different cut-off value of NLR, the impact of potential confounders, and the small number of individual studies. Meanwhile, mounting evidence indicates that a higher NLR on admission is an independent risk factor of sICH^23–25^. Elucidation of the clinical significance of NLR is needed.

Thus, we performed this meta-analysis to comprehensively summarize the prognosis value of elevated NLR level for poor functional outcome, sICH and 3-month mortality in AIS patients under reperfusion therapy.

## Methods

### Search strategy and eligibility criteria

The current study was conducted according to the Preferred Reporting Items for Systematic Reviews and Meta-Analyses guidelines (PRISMA)^26^.

#### Search strategies

We carefully searched PubMed and EMBASE to identify relevant studies published before January 19th, 2019. The search phrases for the two databases included Boolean terms ‘AND’ and ‘OR’ with the following keywords in various possible combinations: “neutrophil to lymphocyte ratio”, “NLR”, “neutrophil”, “lymphocyte”, “recombinant tissue plasminogen activator”, “tissue plasminogen activator”, “rtPa”, “tPa”, “thrombolysis”, “endovascular treatment”, “mechanical thrombectomy”, “Thrombectomy” and “Recanalization”. Reference lists from all included articles, reviews on the topic, and the authors’ own files were also searched for relevant studies. Besides, abstracts from scientific conference were also searched and appraised.

#### Inclusion criteria

Studies were considered eligible for inclusion if they met the following criteria: (1) designed as a cohort study; (2) they evaluated the potential association between baseline NLR levels after AIS onset and the patients’ outcomes after reperfusion therapies including IVT or EVT; (3) NLR was calculated from blood samples collected on admission by the following equation: NLR = Neutrophil counts/Lymphocyte counts; (4) the outcomes for evaluation included therapy related early sICH, 3-month mRS classified as good/poor or 3-month mortality; (5) mean and standard deviation (SD) or median and interquartile range (IQR) of NLR in both good and poor outcome groups were given. Odds ratio (OR) for the outcome of AIS patients after reperfusion therapies was reported or could be calculated from original articles.

#### Exclusion criteria

Exclusion criteria were (any single one was enough for exclusion): (1) the study designed as a review, a case report, a letter or an animal study; (2) studies with insufficient data for extraction.

### Data extraction and quality assessment

Two blinded reviewers (Bi and Chen) independently extracted data from all potentially relevant papers. The extracted data elements of this review included: (1) publication details: first author’s last name, publication year, and origin of the studied population; (2) study design; (3) characteristics of the patients: number of participants, age, gender, sampling time of the blood, NIHSS on admission, follow-up duration; (4) participants number across different outcome groups (good outcome and poor outcome), means and SDs of baseline NLR in each group, or OR for poor outcomes with 95% CI, the cut-off value of NLR and number of participants with the outcomes of interest (early sICH; poor functional outcome, defined as 3-month modified Rankin Score [mRS] > 2 or ≥ 2; and 3-month mortality in each group). The quality assessment of each article was evaluated by the Newcastle–Ottawa Scale (NOS), according to which six or more stars were considered to be of high quality^27^ (Supplementary Table S1). Overall quality of the evidence for NLR level and each outcome meta-analysis was rated using the Grading of Recommendations Assessment, Development, and Evaluation (GRADE) system using GRADEpro Guideline Development Tool (McMaster University, Hamilton, ON, Canada) (Table 1). Any disagreement was resolved by consensus with a third reviewer (Xia YP).

**Table 1:** Qualitative assessment of results of metanalysis for NLR in AIS patients outcome after reperfusion therapy (GRADE analysis).

No of studies	Certainty assessment						No of patients	Effect	Certainty
	Study design	Risk of bias^a^	Inconsistency^b^	Indirectness^c^	Imprecision^d^	Other considerations		Absolute (95% CI)	
**Baseline NLR level and poor functional outcome at 3 months (primary meta-analysis**^**1**^**)**									
6	Observational studies	Not serious	Serious	Not serious	Serious	None	2712	0.54 (0.38 to 0.70)	⨁◯◯◯ VERY LOW
**Baseline NLR level and poor functional outcome at 3 months (secondary meta-analysis**^**2**^**)**									
5	Observational studies	Not serious	Not serious	Not serious	Not serious	None	2436	0.6 (0.49 to 0.71)	⨁⨁◯◯ LOW
**Baseline NLR and the risk of sICH (primary meta-analysis**^**1**^**)**									
4	Observational studies	Not serious	Serious	Not serious	Not serious	None	2003	0.78 (0.18 to 1.37)	⨁◯◯◯ VERY LOW
**Baseline NLR and the risk of sICH (secondary meta-analysis**^**2**^**)**									
3	Observational studies	Not serious	Not serious	Not serious	Not serious	None	1814	1.1 (0.88 to 1.33)	⨁⨁◯◯ LOW
**Baseline NLR and 3-month motality**									
3	Observational studies	Not serious	Not serious	Not serious	Not serious	None	1389	1.71 (1.01 to 2.42)	⨁⨁◯◯ LOW

*CI* confidence interval.

^a^Risk of bias: based on Newcastle-Otawa Scale for cohort studies and blind assay assessment reported.

^b^Inconsistency: based on the evaluation of heterogeneity (Q statistic and I2 statistic) in this meta-analysis.

^c^Indirectness: refers to how well the evidence included in the review answers the review question related to population, intervention, comparator or outcome used in included studies.

^d^Imprecision: results are considered imprecise when studies include only relatively few participants (a total number of participants is less than 400 for continuous outcome information is considered to be insufficient) and when 95% confidence interval include no effect and the upper confidence limit cross the minimal important difference.

^1^Primary meta-analysis pooled all the studies comprising the outcome into analysis.

^2^Secondary meta-analysis excluded one study which brought high heterogeneity before analysis.

### Statistical analyses

Data were pooled in a meta-analysis when at least 2 studies with relevant data were available. For studies using ORs for risk estimates, we uniformly transformed them into ORs which evaluated the effect of increased NLR level on poor outcome. To accommodate differences in the way in which NLR measured, the absolute NLR levels were converted into a common unit by calculating standardized mean difference (SMD) for articles reporting NLR as a continuous variable. If the study provided medians and IQR but not means and SDs, we estimated the means and SDs using the median and the estimator SD = IQR/1.35^28^.

In all analyses, we used a random effects model. We quantified the strength of the association between NLR level and poor functional outcome (3-month mRS > 2 or ≥ 2) or sICH after reperfusion therapy using SMD. The cumulative risk of 3-month mortality in patients of higher NLR level was calculated both from SMD and OR and their corresponding 95% CI. We assessed statistical heterogeneity using I-squared statistics and Cochran Q test, sensitivity and subgroup analyses were performed to explore sources of heterogeneity. Possible publication bias was evaluated by constructing a funnel plot. We assessed funnel plot asymmetry using Begg’s and Egger’s tests and defined significant publication bias as p value < 0.1. All analyses were conducted by StataSE12.

## Results

### Search results

**Figure 1:**
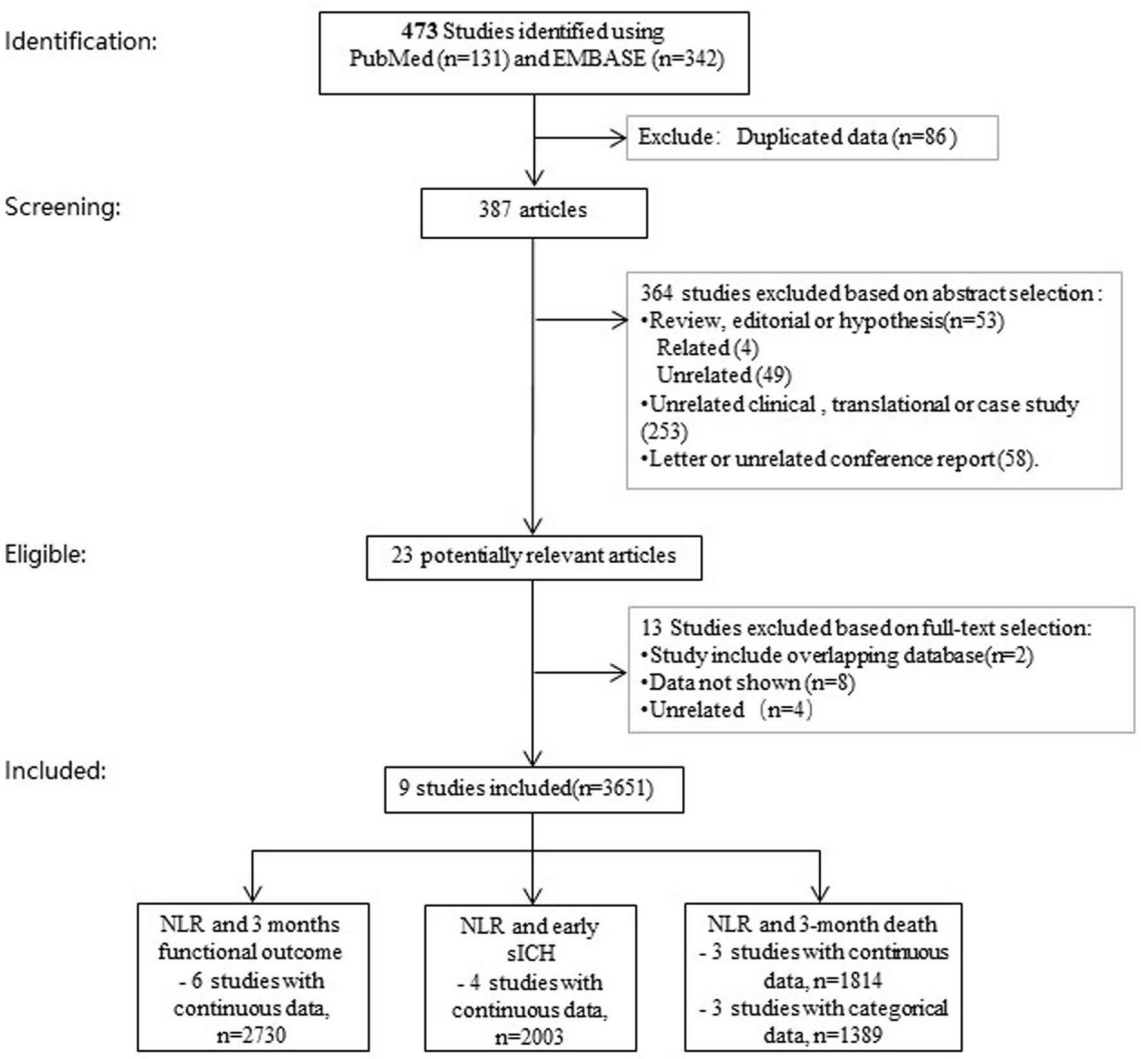
Flow chart of literature search and study selection. *NLR* neutrophil to lymphocyte ratio, *ICH* intracranial hemorrhage.

The literature research initially retrieved 473 studies. Among them, 86 were duplicated data and were then removed. 364 studies were excluded after screening for the titles and abstracts. 14 studies were removed after 2 reviewers independently read the full text and determined that those studies did not meet inclusion criteria. Consequently, nine studies including a total of 3651 participants met our inclusion criteria and were pooled in meta-analysis^21–25,29–32^ (Fig. 1).

### Characteristics of included studies

A summary of the characteristics of included studies and quality indicators were depicted in Table 2. The 9 studies were all published between 2014 and 2018^21–25,29–32^. The method of reperfusion therapy was IVT in 4 studies and EVT in other 5 researches. The NLR level in all of the included studies was the baseline level on admission before reperfusion therapy. Poor functional outcome was defined as mRS > 2 in 7 studies, mRS ≥ 2 in 2 studies, and mRS > 3 in one study. sICH was reported in 4 studies, among which only one explored the relationship of NLR level and increased risk of parenchymal hematomas^32^.

**Table 2:** Main characteristics of studies included in the meta-analysis.

Author	Year	Study location	Study design	Reperfusion treatment	Time to Lb test	NLR cut-off value	Patients, n	Mean Age, years	Male, %	NIHSS on admission, mean or median	Fellow-up	Outcome	NOS
Malhotra et al.	2018	The USA	Retrospective	IV tPA	On admission	2.2	657	64.3	50.7	7 ( 4–13)*	3 months	mRS (0–1) vs (2–6), mRS (0–2) vs (3–6), 3-month mortality, and sICH	6
												sICH: within 22–36 h after treatment, SITSMOST definition	
Wang et al.	2018	China	Retrospective	EVT, prior IVT or not	On admission	NR	199	64*	60.2	16 (13–21)*	3 months	mRS(0–2) vs (3–6) and sICH	7
												sICH: within 72 h after treatment, defined by the criteria of the HBC	
Goyal et al.	2018	The USA	Retrospective	MT, prior IVT or not	On admission	NR	293	62	50	16 (13–19)*	3 months	mRS (0–2) vs (3–6), 3-month mortality, and sICH	6
												sICH:within 36 h after treatment, SITSMOST definition	
Duan et al.	2018	China	Retrospective	Bridging therapy (IVT + EVT), or direct EVT	Prior to EVT	7	616	66*	59.7	16(12–21)*	3 months	mRS (0–2) vs (3–6), 3-month mortality, and sICH	7
												sICH:within 72 h after treatment, defined by the criteria of the HBC	
Pagram et al.	2016	Australia	Retrospective	IV rtPA	Prior to thrombolysis and 24 h after stroke	NR	142	74.3	NR	14.1	3 months	mRS (0–2) vs (3–6)	7
Guo et al.	2016	China	Prospective	IVT or bridging therapy (IVT followed by EVT)	On admission and 12-18 h after IV rtPA	NR	189	65	65.1	12(6–16)*	48 h	PH and sICH	8
												sICH:within the first 7 days, the ECASS-II definition	
Semerano et al.	2016	Italy	Retrospective	IVT	Within 48 h from symptom onset	NR	575	NR	NR	NR	3 months	mRS (0–2) vs (3–6) and sICH	5
												sICH(NR)	
Maestrini et al.	2015	Finland	Retrospective	IV rtPA	On admission	4.8	864	71*	50.8	10(6–16)*	3 months	mRS (0–1) vs (2–6), mRS (0–2) vs (3–6), 3-month mortality, and sICH	6
												sICH:within the first 7 days, the ECASS-II definition	
Brooks et al.	2014	The USA	Retrospective	IV rtPA, IA rtPA, or MT	On admission	5.9	116	67	NR	17	3 months	mRS (0–3) vs (4–6) and 3-month mortality	7

*Lb* laboratory, *IV* intravenous, *IVT* intravenous thrombolysis, *EVT* endovascular treatment, *MT* mechanical thrombectomy, *IA* Intra-artery, *SITSMOST* Safe Implementation of Thrombolysis in Stroke-MOnitoring Study, Lumbar Disc Herniation, *HBC* Heidelberg bleeding classification, *the ECASS-II definition* the European Cooperative Acute Stroke–II definition, *PH* parenchymal hematomas, *NR* not reported.

*Median, Confidence interval.

OR value was applied for prognostic evaluation in 4 studies. NLR level was presented as a categorical variable in these 4 studies and the cut-off value were: 2.2, 7.0, 4.8 and 5.9^21–24^. NLR level was analyzed as a continuous variable in 7 studies. 2 studies reporting NLR level in both variable types^23,24^. The quality assessment of the 9 studies was presented in Table 2. Not all studies provided the same range of outcome measures. Consequently, different total number of patients contributed in each meta-analysis.

### Baseline NLR level and 3-month poor functional outcome

**Figure 2:**
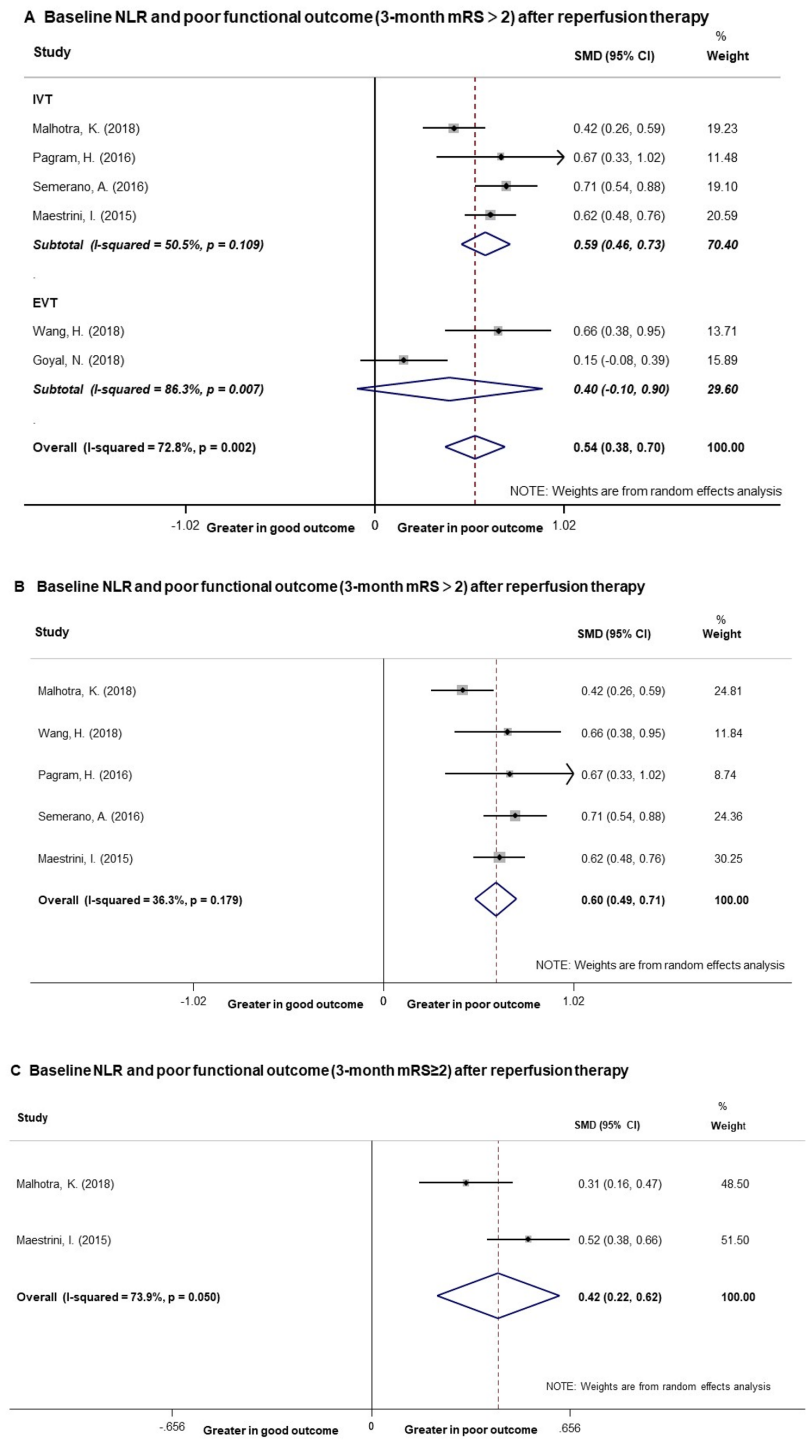
Forest plots of baseline NLR level and poor functional outcome at 3 months. (**A**) Pooled analysis of poor functional outcome (defined as mRS > 2) at 3 months after reperfusion therapy. (**B**) Pooled analysis of poor functional outcome (defined as mRS > 2) at 3 months after reperfusion therapy after excluding one study which brought high heterogeneity. (**C**) Pooled analysis of poor functional outcome (defined as mRS ≥ 2) at 3 months after reperfusion therapy in AIS patients. *NLR* Neutrophil to lymphocyte ratio, *mRS* modified Rankin Score.

6 studies including 2730 patients provided data on functional outcome defined as mRS > 2^23–25,29–31^. Overall, pooled analysis of the 6 studies showed that baseline NLR level was greater in patients with 3-month mRS > 2. The SMD in the NLR levels between the patients with mRS > 2 and those with mRS ≤ 2 was 0.54 units (95% CI [0.38, 0.70]) (Fig. 2A), and the z-score for overall effect was 6.56 (p < 0.05). The heterogeneity test showed that there were significant differences between individual studies (p = 0.002; I^2^ = 72.8%).

**Figure 3:**
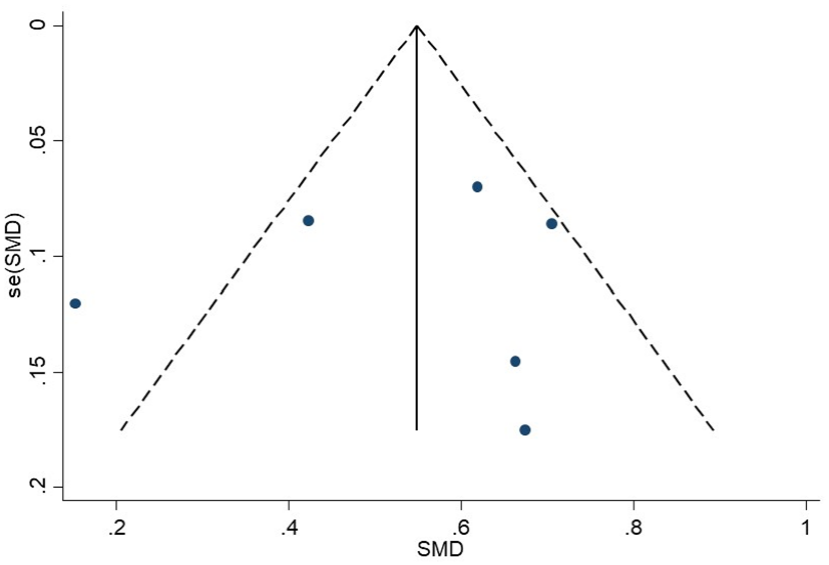
Funnel plot of the meta-analysis between baseline NLR level and poor functional outcome (mRS > 2) at 3 months. *NLR* neutrophil to lymphocyte ratio, *mRS* modified Rankin Score.

No publication bias was evident (p = 0.78) measured by funnel plot, Begg’s and Egger’s test (Fig. 3). To find out the origin of heterogeneity, we subsequently performed subgroup analyses according to different reperfusion therapies of the studies as illustrated in Fig. 2A. The pooled SMD was 0.59 (95% CI [0.46, 0.73]) and I^2^ reduced to 50.5% for the 4 studies with IVT treatment. As to the two studies with EVT treatment, the pooled SMD was 0.40 (95% CI [− 0.10, 0.90], I^2^ = 86.3%) with no significant difference. We performed sensitivity analyses to find the origin of heterogeneity. After removing the study by Goyal et al.^25^ that enrolled patients presenting between 6 and 12 h after symptom onset, the analysis did not find significant influence on the result (Fig. 2B). Of note, after excluding the study, heterogeneity test showed that there were less significant differences between the remaining 5 studies (I^2^ = 36.3%, p = 0.179) which included AIS patients presenting within 6 h. Therefore, differences in the presenting time may be a possible source of heterogeneity.

2 of the 6 studies including 1521 patients also reported data on functional outcome defined as mRS ≥ 2^23,24^. Pooled analysis demonstrated that patients with poor functional outcome (mRS ≥ 2) had greater baseline NLR levels than those with good functional outcome (mRS < 2) (SMD = 0.42, 95% CI = [0.22, 0.62], I^2^ = 73.9%) (Fig. 2C).

### Baseline NLR level and sICH

**Figure 4:**
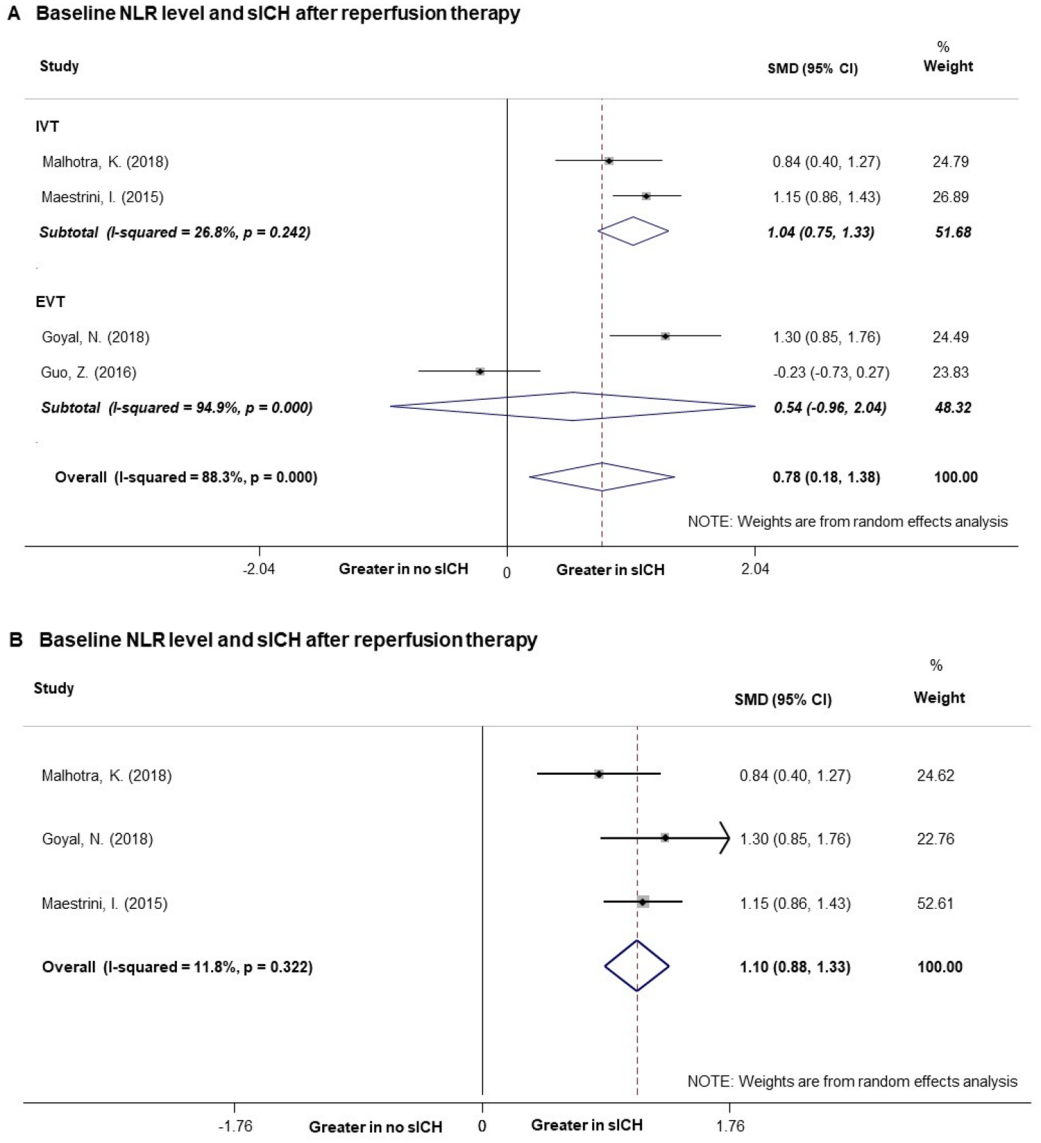
Forest plots of baseline NLR level and risk of early symptomatic intracerebral hemorrhage. (**A**) Pooled analysis of sICH after reperfusion therapy. (**B**) Pooled analysis of sICH after reperfusion therapy after excluding one study which brought high heterogeneity. *NLR* Neutrophil to lymphocyte ratio, *sICH* symptomatic intracerebral hemorrhage.

Three studies showed that patients with sICH after reperfusion therapies had greater baseline NLR levels^23–25^, whereas the NLR levels did not show significant difference between the 2 groups in one other study^32^. Overall, NLR levels were greater in patients with sICH occurrence in our study (Fig. 4A). The SMD in the NLR levels between the patients with and those without occurrence of sICH was 0.78 units (95% CI [0.18, 1.38]). The heterogeneity test showed significant differences between individual studies (p < 0.001; I^2^ = 88.3%). No significant publication bias was found in the meta-analysis (p = 0.416). In subgroup analyses including only patients treated by IVT, baseline NLR level was associated with sICH occurrence (SMD = 1.04, 95% CI [0.75, 1.33]) and the heterogeneity reduced to 26.8% (p = 0.242). Sensitivity analysis indicated that the study of Guo et al.^32^, account for the high heterogeneity of the 4 studies that pooled into analysis. The study included patients treated with IVT or a bridging therapy consisting of IVT followed by endovascular therapy. However, the treating time of bridging therapy was not mentioned, which has found to be a strong influencing factor of patients’ outcome. Besides, this study was a prospective cohort study, different form other 3 retrospective study design. Both could be the possible source of heterogeneity. After excluding the study, heterogeneity test showed that there were low significant differences between the remaining 3 studies (I^2^ = 11.8%, p = 0.322) and showed no significant influence on the results (Fig. 4B). Further exclusion of any single study with sICH did not significantly alter the pooled SMD and heterogeneity.

### Baseline NLR level and 3-month mortality

**Figure 5:**
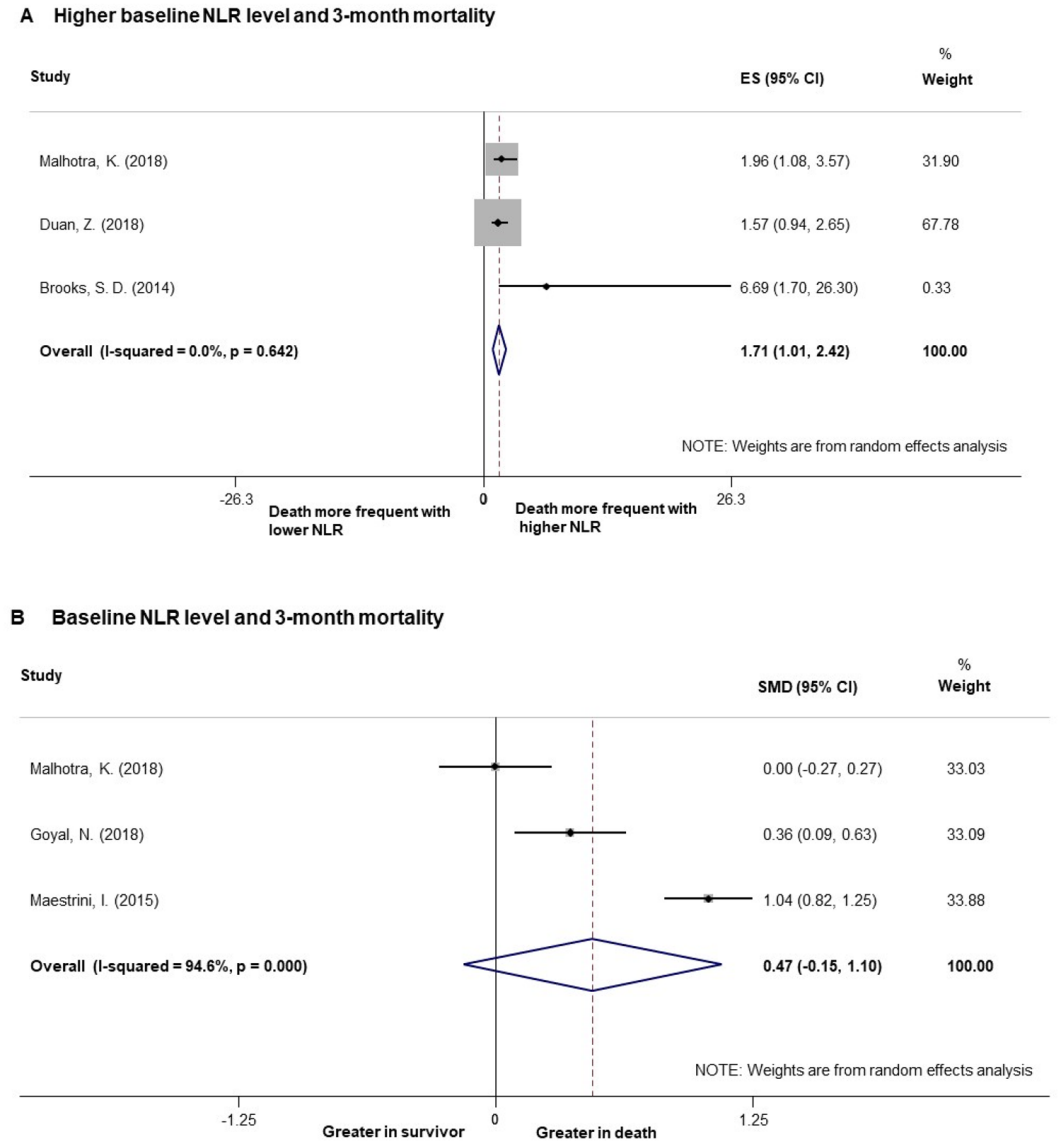
Forest plots of baseline NLR level and 3-month mortality. (**A**) Pooled analysis of 3-month mortality with studies regarding NLR as binary data. (**B**) Pooled analysis of 3-month mortality with studies regarding NLR as continuous data. *NLR* neutrophil to lymphocyte ratio.

There were 5 studies reporting the relationship between baseline NLR level and 3-month mortality. 2 of them regarded NLR level as a categorical variable, other two regarded it as a continuous variable and one provided both forms of data. Pooled analysis using categorical variable from the above 3 studies demonstrated the odds of 3-month mortality in AIS patients with reperfusion therapy to be significantly elevated in those with higher NLR levels (ES = 1.71, 95% CI [1.01, 2.42], p < 0.01, I^2^ = 0%). However, pooled analysis of the 3 studies using NLR level as a continuous variable found no significant difference between 3-month death and survivor (Fig. 5).

## Discussion

Our results show that, in AIS patients with reperfusion therapy, increased baseline NLR level is associated with 3-month poor functional outcome and the risk of sICH. In addition, we suppose that higher NLR level is associated with 3-month mortality, consistent with previous studies highlighting that NLR is related with mortality in the general population and long-term major cardiovascular events^33,34^. These data thus suggest high baseline NLR level as a predictor for poor outcome in AIS patients treated with reperfusion therapy.

Reperfusion therapy is becoming more feasible in clinical practice with the progress of health education and neurologist’s attention. Even though technical improvements in reperfusion therapy have resulted in significantly higher rate of revascularization and improved clinical outcomes in AIS, morbidity and mortality remain considerable in large vessel intracranial occlusion patients despite MT^35–37^. Meanwhile, pooled analyses showed that tPA increased hemorrhagic transformation especially when administered from 3 to 4.5 h after onset of symptoms^38,39^. Even within 4.5 h of stroke onset, early treatment is very important for patients with severe stroke because of the increasing risk of sICH. Therefore, the prediction of sICH and poor outcomes after reperfusion therapies might guide early prevention and increase the number of patients who would benefit most from reperfusion.

Recently, predictors have been discovered to indicate outcome of reperfusion therapy in AIS patients, including imaging examination reports^40,41^, hematological examination results such as HbA1c (glycated hemoglobin) levels and serum levels of caveolin-1^42,43^, and the baseline NIH Stroke Scale^44^. However, there is still a lack of indicators which are widely accessible and usually collected in routine medical practice. Neutrophils and monocyte count^45,46^, widely available, were both found to predict 3-month outcome of reperfusion therapy. NLR level was a promising predictor that was well studied in cardiac revascularization and cerebral vascular disease including AIS and cerebral hemorrhage^11,19–22,47^. Interestingly, admission NLR was found to be a possible marker for distinguishing between atherothrombotic and cardioembolic ischemic strokes^48^. This manuscript pooled most recent studied into meta-analysis and suggests NLR as an outcome predictor for AIS patients under reperfusion therapy. Easily available and cost-effective, NLR would be a promising prognostic factor to be used routinely in clinical practices and guide the early prevention of bad outcomes. If patients with high NLR level receive EVT, it is essential to intensify monitoring and care after operation and to increase communication with the family about patient’s condition. More neuroprotection strategies are needed for patients with high admission NLR. We cannot recommend using NLR alone for patient selection for revascularization treatment because it will increase delays in all stroke patients for a not yet proven benefit. Notably, NLR might be one of the variables in various scoring tools for risk benefit judgment and to facilitate patient selection. One of the individual components of PREDICT scale is admission NLR^29^. Besides, studies are needed to approve whether NLR would be a variable considered for low-dose rtPA when thrombolysis in severe stroke patients.

Shi, R suggested that immediately after ischemic stroke onset, there was an exponential increase in the neutrophil count and an exponential decrease in the lymphocyte count^49^, consistently with the preceding results. The hypothalamo-pituitary-adrenal (HPA) axis, the sympathetic–adrenal–medullary (SAM) axis and the para sympathetic nervous system (vagus nerve) are involved in immune systems regulation the moment stroke onsets^50^. Accelerated apoptosis of lymphocytes are observed and associated with increased catecholamines level, which lead to lymphocytopenia^51–53^. Neutrophils in bone marrow are stimulated by growth factors and chemokines that facilitate neutrophil trafficking within the first 24 h after symptom onset, contributing to peripheral blood neutrophils elevating^54,55^.

Evidenced on this, there are several possible explanations for the relationship between NLR and poor functional outcomes of reperfusion therapy in AIS patients. On the one hand, studies approved that higher neutrophil level predicted detrimental stroke outcomes. The elevation of neutrophils was positively correlated with NIHSS scores and infarct sizes, which may partly be related with neutrophil extracellular trap formation and proinflammation cytokines secretion^53,56^. On the other hand, lower level of lymphocytes was found related with poor outcome. Lymphocytopenia and post-stroke immunodeficiency promotes spontaneous bacterial infections, such as stroke-associated pneumonia or intestines infection^57^.

Besides, neutrophils could synthetize and express MMP-9 as well as inter cellular adhesion molecule1(ICAM-1). Thus, high level of neutrophils may increase hemorrhagic transformation risk after tPA treatment as well as MT^56^. Platelets could seal the damaged vessels and reduce the leakage of blood to the ischemic brain parenchyma in AIS patients. Lymphocytes mediated this phenomenon by interacting platelets through P-selectin glycoprotein ligand-1 (PSGL-1)^58^. Therefore, low level of lymphocytes could favor hemorrhage transformation especially in large infarctions. Collectively, NLR may enhance the predict value of both neutrophil count and lymphocyte count respectively. Actually, Cai et al. suggested that NLR was positively correlated with higher NIHSS and infarct sizes based on a case–control study including 225 AIS patients and 56 age-and gender matched healthy controls^54^. Nam et al. found that high NLR predicted stroke-associated pneumonia in AIS patients, which would exacerbate further functional outcome or even lead to death^59^. Both findings partly support our result that NLR is related with poor functional outcome after reperfusion therapy in AIS patients.

There are several strengths and limitations to consider in this study. One of the strengths is the inclusion of cohort study design in all the included studies. Moreover, the studies were included in the final analysis based on clear inclusion and exclusion criteria. In addition, to the best of our knowledge, no meta-analysis on this topic has been conducted and our study adds to the current understanding of the association between inflammation and outcomes of reperfusion therapies after AIS onset. However, some potential limitations may be apparent. Firstly, our analysis is based on observational studies, most of which were retrospective, and may be subjected to the potential biases of such studies. Secondly, converting abnormally distributed statistics to normally distributed statistics may be a source of bias in our analysis. While the direction of effect estimate is likely to be accurate, the large I^2^ statistics and low GRADE scores in many analyses limits the conclusions that can be drawn from the results. Therefore, our results should be interpreted cautiously. Finally, this meta-analysis was not registered in advance and giving that the number of studies in our meta-analysis was small, we cannot exclude the possibility of publication bias, although our funnel plot showed that publication bias is unlikely^60^.

## Conclusion

In conclusion, our meta-analysis demonstrates that, for AIS patients, increased baseline NLR level may predict 3-month outcome and higher risk of sICH occurrence after reperfusion therapies. NLR level can be eventually incorporated into routine clinical practice for patient prognostication and risk stratification after further explored by larger well-designed studies.

## Supplementary Information

Supplementary Table S1.

Supplementary Figures.
