# Supplementary material for: Prognostic value of neutrophil to lymphocyte ratio in acute ischemic stroke after reperfusion therapy
Source: Sci Rep. 2021 Mar 17;11:6177. doi: 10.1038/s41598-021-85373-5 (PMC7971057; doi:10.1038/s41598-021-85373-5)
Supplement: Supplementary file 1 — Supplementary Table S1. [file 41598_2021_85373_MOESM1_ESM.docx]

**P****rognostic value of neutrophil to lymphocyte ratio in acute ischemic stroke after reperfusion therapy**

Ying Bi**^#^**, Jing Shen, Sheng-Cai Chen, Ji-Xiang Chen*****, Yuan-Peng Xia*****

Department of Neurology, Union Hospital, Tongji Medical College, Huazhong University of Science and Technology, Wuhan 430022, China.

***Correspondence:** Ji-Xiang Chen, Yuan-Peng Xia

xiayuanpeng@hust.edu.cn.

| **Supplementary Table S1. The Newcastle-Ottawa Scale (NOS) assessment of each article included in the meta-analysis** | | | | | | | | |  |
| --- | --- | --- | --- | --- | --- | --- | --- | --- | --- |
| **Study** | **Selection** | | | | **Comparability** | **Outcome** | | | **NOS** |
|  | Representativeness of the exposed cohort | Selection of the non exposed cohort | Ascertainment of exposure | Demonstration that outcome of interest was not present at start of study | Comparability of cohorts on the basis of the design or analysis | Assessment of outcome | Was follow-up long enough for outcomes to occur | Adequacy of follow up of cohorts |  |
| Malhotra, K. 2018 | * | * | * | - | - | * | * | * | 6 |
| Wang, H. 2018 | * | * | * | - | * | * | * | * | 7 |
| Goyal, N. 2018 | * | * | * | - | - | * | * | * | 6 |
| Duan, Z. 2018 | * | * | * | - | * | * | * | * | 7 |
| Pagram, H. 2016 | * | * | * | - | * | * | * | * | 7 |
| Guo, Z. 2016 | * | * | * | * | * | * | * | * | 8 |
| Semerano, A. 2016 | * | * | * | - | - | * | * | - | 5 |
| Maestrini, I. 2015 | * | * | * | - | - | * | * | * | 6 |
| Brooks, S. D. 2014 | * | * | * | - | * | * | * | * | 7 |
