# Supplementary material for: Prognostic value of neutrophil to lymphocyte ratio in acute ischemic stroke after reperfusion therapy
Source: Sci Rep. 2021 Mar 17;11:6177. doi: 10.1038/s41598-021-85373-5 (PMC7971057; doi:10.1038/s41598-021-85373-5)
Supplement: Supplementary file 2 — Supplementary Figures. [file 41598_2021_85373_MOESM2_ESM.docx]

**P****rognostic value of neutrophil to lymphocyte ratio in acute ischemic stroke after reperfusion therapy**

Ying Bi**^#^**, Jing Shen, Sheng-Cai Chen, Ji-Xiang Chen*****, Yuan-Peng Xia*****

Department of Neurology, Union Hospital, Tongji Medical College, Huazhong University of Science and Technology, Wuhan 430022, China.

***Correspondence:** Ji-Xiang Chen, Yuan-Peng Xia

xiayuanpeng@hust.edu.cn.


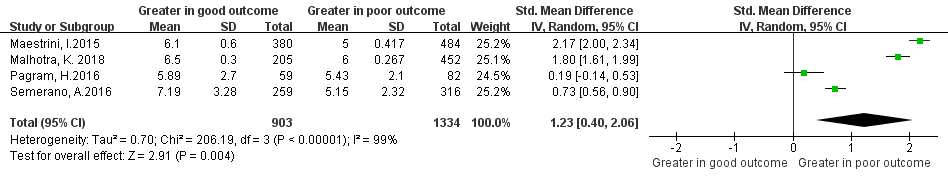


Supplementary Figure S1. Forest plot of baseline Neutrophil level and pool functional outcome at 3 months


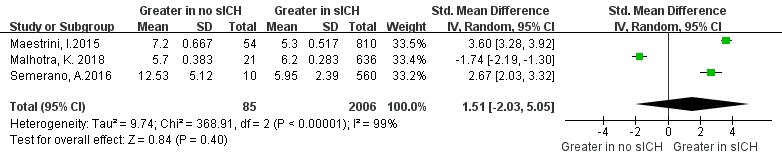


Supplementary Figure S2. Forest plot of baseline Neutrophil level and risk of early symptomatic intracerebral hemorrhage


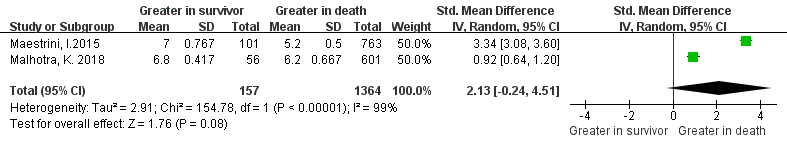


Supplementary Figure S3. Forest plot of baseline Neutrophil level and 3-month mortality
